# Proactive and Reactive Language Control in the Bilingual Brain

**DOI:** 10.3390/brainsci9070161

**Published:** 2019-07-10

**Authors:** Roy Seo, Chantel S. Prat

**Affiliations:** 1 Department of Psychology, University of Washington, 119A Guthrie Hall, UW Box 351525, Seattle, WA 98195, USA; 2 Institute for Learning and Brain Sciences, University of Washington, 1715 Columbia Road N., Portage Bay Building, Box 357988, Seattle, WA 98195, USA

**Keywords:** bilingual language control, proactive control, reactive control, cognitive control, anterior cingulate cortex, basal ganglia, dorsolateral prefrontal cortex

## Abstract

The current experiment investigated bilingual language control within the dual mechanisms framework. In an fMRI investigation of morphosyntactic rule production, the presence or absence of target language cues was manipulated to investigate the neural mechanisms associated with proactive and reactive global language control mechanisms. Patterns of activation across nine regions of interest (ROIs) were investigated in seventeen early Spanish–English bilingual speakers. A cue by phase interaction in the left dorsolateral prefrontal cortex (DLPFC) and pre-supplementary motor area (Pre-SMA) was observed, suggesting that these regions were more active during cue phases, and less active during execution phases, when target language cues were presented. Individual differences analyses showed that variability in proactive control (informative > non-informative cued trial activation during preparation) in the basal ganglia was correlated with proactive control in the left DLPFC, left inferior frontal gyrus (IFG), and right precentral ROIs. In contrast, reactive control (non-informative > informative cued activation during execution) in the anterior cingulate was correlated with reactive control in the Pre-SMA and left orbital frontal ROIs. The results suggest that, consistent with the dual mechanisms framework, bilinguals differ in the degree to which they use cues to proactively prepare to use a target language.

## 1. Introduction

The average speaker knows more than one language [1,2], and an increasing amount of research has been dedicated to understanding the particular neurocognitive demands associated with managing multiple languages [1,3,4,5,6]. These demands arise because relevant information becomes activated across languages, creating additional requirements for selection and interference management processes [6,7,8,9,10].

Research on bilingual language control has increasingly highlighted its dynamic and adaptive nature. For instance, bilinguals must deploy control mechanisms across different levels of selection such as globally biasing the availability of one language over another versus selecting among local competitors at the morphosyntactic and lexico-semantic levels [3]. The former is unique to bilingual language control and the latter process is shared between bilinguals and monolinguals. Additionally, bilingual language control processes can be deployed ahead of time, or proactively, under conditions in which an individual knows which language he or she will need to use in advance, or “on the fly,” or reactively, when linguistic cues are not provided [3,11].

One of the most influential models of the neurocognitive basis of bilingual language control is the adaptive control hypothesis [11], which posits that bilinguals deploy a network of regions more generally involved in action selection and cognitive control to manage competition between languages. One of the central tenets of this hypothesis is that bilingual language control, like cognitive control more generally, changes according to the demands of the linguistic environment in which a bilingual is speaking. Specifically, the adaptive control hypothesis describes eight control mechanisms: goal maintenance, conflict monitoring, interference suppression, salient cue detection, selective response inhibition, task engagement, task disengagement, and opportunistic planning, and the bilingual contexts under which each process would most likely be engaged [11]. Green and Abutalebi also propose a network of regions involved in these processes including the anterior cingulate cortex (ACC) and pre-supplementary motor area (Pre-SMA) for conflict monitoring and speech control, the left inferior frontal (IFG) and dorsolateral prefrontal cortex (DLPFC) for interference suppression, bilateral parietal lobes for goal maintenance pertaining to the language in use, right inferior frontal cortex and thalamus for salient cue detection, response inhibition, and the left caudate nucleus for executing language switching [3,11].

Although the adaptive control hypothesis accounts for many phenomena described in the bilingual language control literature, its emphasis is on changes that occur based on the differing demands of bilingual language environments. Much less research or theory has been devoted to understanding individual differences reflected by bilinguals operating under the *same* linguistic pressures. Take, for example, the dual mechanisms framework described by Braver and colleagues [12] which is related to overlapping cognitive control constructs outlined in the adaptive control hypothesis.

Specifically, the dual mechanisms framework proposes two modes in which cognitive control processes might be deployed: proactive control, a form of early selection or attention biasing during which information about a task is maintained in working memory and used to optimally guide subsequent perception and action systems, and reactive control, which is recruited “on the fly” as a late correction, when a high-conflict event is detected. Importantly, the dual mechanisms framework highlights that the deployment of these two types of control differs both as a function of the characteristics of the task (e.g., whether or not an opportunity to prepare is provided) and the characteristics of an individual (e.g., whether or not an individual has sufficient working memory capacity to maintain a goal in mind). In the current investigation, we argue that failure to consider such individual differences may lead to some inconsistencies in the bilingual language control literature.

For example, one meta-analysis of neuroimaging research on bilingual language control did not find consistent involvement of either the ACC or the parietal cortices, which are central to the adaptive control hypothesis [13]. Additionally, the meta-analysis revealed reliable involvement of the left inferior-orbital frontal region (BA47), the right caudate nucleus, and bilateral temporal regions, which are not included in the adaptive control hypothesis. The nature of these regions’ contributions to bilingual language control may be related to individual differences in deployment of proactive and reactive control processes. Specifically, according to the dual mechanisms framework [12], proactive control requires goal maintenance, which is underpinned by early and sustained activation of the lateral prefrontal cortices, accompanied by dopaminergic responses to contextual (salient) cues. Adaptive control proposes that goal maintenance in bilingual language control is accomplished by another region associated with working memory, the parietal lobes. In either case, an increasing body of research suggests that not all individuals engage in proactive control, even when given the chance to do so [14,15]. Furthermore, Braver discusses reactive control as being underpinned by transient lateral prefrontal activation triggered either by the ACC conflict monitoring system or by a temporal associative memory region [12]. Again, the extent to which an individual relies upon reactive control is inversely related to their early deployment of proactive mechanisms. This consideration offers both a possible explanation for the role of the medial temporal regions uncovered by the meta-analysis, and a potential explanation for why ACC contributions to bilingual language control were not consistent enough to be uncovered by the meta analysis.

Understanding the implications of individual differences, as proposed by the dual mechanisms account of cognitive control, is important, as an increasing body of behavioral and EEG research has demonstrated that bilinguals deploy proactive control mechanism when cues (either from the environment or the experimental paradigm) provide them with information about which language they will be speaking in [16,17,18,19]. For instance, the impact of target language cueing was recently studied by Grainger and colleagues who showed that French–English bilinguals’ performance on a bilingual lexical decision task was significantly facilitated when an image of a French or British flag preceded the word or pseudoword by 50 ms. Importantly, this facilitation was only observed when the flag and language matched (e.g., a British flag preceded an English word) [17]. Related evidence was provided by Woumans and colleagues who trained Spanish–Catalan and Dutch–French bilingual participants to associate specific interlocutors’ faces with predictable speaking behaviors [19]. Through simulated Skype interactions, each of twelve faces was associated with speaking only one of the bilinguals’ two languages. At test, bilingual participants completed a verb generation study in which they were asked to produce a verb related to a noun presented in one of their two languages. When a trial was preceded (2000 ms) by a previously trained face, response times were facilitated over conditions in which a non-familiar face was presented. This facilitation was limited, initially, to congruent conditions in which the language spoken by the familiar face matched the language in which the trial was presented. Together, both studies demonstrated that bilinguals use proactive cues to facilitate language control, showing that *informative* cues resulted in shorter response times during various language comprehension and production processes. 

To date, only a few experiments have investigated the neurobiology of proactive control mechanisms in bilinguals. In a recent experiment, Seo and colleagues [20] investigated patterns of activation in nine ROIs previously identified by either the meta-analysis [13], or by the adaptive control hypothesis [11] using a morpho-syntactic rule execution paradigm. Specifically, to investigate the nature of proactive global and local language selection processes, they employed a Rapid Instructed Task Learning (RITL) paradigm. The RITL paradigm was originally developed to study cognitive flexibility, particularly as it pertains to executing rule-based behaviors [20,21,22]. A critical feature of the RITL paradigm is that the conditions for completing each trial change across the paradigm, which allows one to study dynamic reconfiguring of control structures such as those described in the adaptive control hypothesis. Because the rules are presented before the stimuli on which the rules need to be applied, one can estimate the neural basis of proactive, top-down control structures separately from those involved in more reactive, task execution processes. As such, non-linguistic RITL paradigms have previously been used to explore potential differences in cognitive control structures between monolinguals and bilinguals [23,24,25]. 

**Figure 1:**
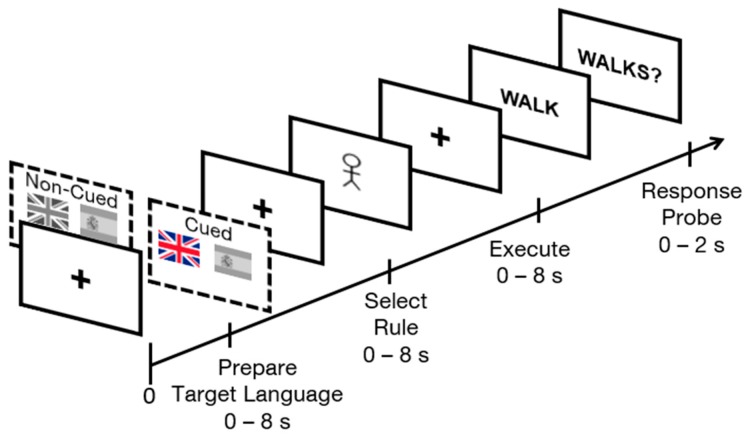
Schematic of sample informative cued and non-informative cued trials in Rapid Instructed Task Learning (RITL) displaying Preparing Target Language, Select Rule, Execute and Response Probe phases. In informative cued trials, one of the two national flags (Great Britain and Spain) is colored, indicating a target language while in non-informative cued trials, both flags are in grey.

Seo et al. [20] were the first, however, to use a RITL paradigm to investigate the role of cognitive control areas *during* bilingual language control [20]. To do so, the rules employed in the RITL task were linguistic in nature. Specifically, trials began with a Prepare Target Language phase, in which a symbolic cue instructed participants which language the trial would be subsequently executed in (e.g., * = English). Then an Encode Rule phase presented a grammatical rule using alphanumeric symbols (e.g., A = pluralize). Finally, during an Execution phase, participants were presented with the linguistic stimuli on which the instructed rule was to be executed (e.g., “dog”), and were asked to sub-vocally produce the resulting transformation. Afterward, they were given a verification probe in the form of a target word or words (e.g., “dogs”), and were asked whether it matched the subvocal manipulation they executed based on the given rule sequence and linguistic input (see Figure 1) [20]. 

The results of the experiment showed three distinct patterns of activation across task phases: presentation of the target language recruited a distributed network including the ACC/Pre-SMA, the left lateral prefrontal cortex, the right precentral gyrus, bilateral caudate nuclei, and bilateral temporo-parietal regions. More focal patterns of activation were observed during local, rule-preparation processes, including the left inferior and middle frontal regions, left parietal regions, the right precentral gyrus, and bilateral caudate nuclei. Region of interest analyses showed that the ACC was primarily activated during top-down global language preparation, which is somewhat inconsistent with both Braver’s dual mechanisms framework and the adaptive control hypothesis [11]. In contrast, activation in three left lateral ROIs as well as in the Pre-SMA increased across task phases and was highest during execution of the morphosyntactic rule. Activity in bilateral caudate nuclei, the middle temporal lobe, and the right precentral gyrus remained active across task phases.

One limitation of using this research to adjudicate between the dual mechanisms and adaptive control accounts is that according to Braver, individuals vary in the extent to which they use cues proactively to prepare for subsequent conflict [26]. Because Seo and colleagues [20] did not have any non-informative cued trials to use as a baseline, and because target language information could eventually be gleaned when the words were given during task execution, the extent to which an individual employed language cues to prepare proactively could not be estimated. In addition, the absence of non-cued trials in the original experiment prevented the researchers from demonstrating the expected behavioral effects of proactive control, namely, that execution was facilitated by the top-down selection of target-language-relevant information. In fact, a subsequent behavioral study by Seo and Prat [25] used a RITL paradigm to show that bilinguals use target language information to globally inhibit information from non-target-language grammatical rule sets, which likely facilitates performance. Another limitation of the previous study is that the cues used to indicate target language and rule instructions were highly arbitrary (numbers and symbols) and participants had to be trained to retrieve the associated rules ahead of time. Thus, the involvement of lateral prefrontal and temporo-parietal regions in the previous study may reflect additional memory demands of the task, rather than any particular process related to bilingual language control. 

The current study aims to circumvent the limitations of existing research in multiple ways. First, like Woumans et al. [19], but unlike Seo et al. [20], the current fMRI study includes conditions in which trials are preceded by either informative (cued) or non-informative (non-cued) indicators of the target language to-be-used. Second, to decrease the memory demands associated with remembering abstract symbol-rule combinations, symbols with significant pre-existing semantic content (e.g., national flags representing the countries that share names with the languages and one versus two stick figures to represent singular versus plural verb forms) were used to indicate the rules. Third, to investigate the difference between global language preparation and global language selection or inhibition the number of trials on which language used switched from one trial to the next (50%) versus remained stable (50%) was manipulated. Finally, the addition of cued and non-cued trials allows us to investigate individual differences in the extent to which individuals employ proactive and reactive control mechanisms during bilingual language control. Such individual differences are central to the dual mechanisms of control theory, but have not yet been systematically investigated in bilingual language control. Importantly, because the primary goal of this study is to integrate the findings from our previous research within the broader literature on bilingual language control by comparing the differing predictions of the adaptive control hypothesis and dual mechanisms theory, we employed a region of interest (ROI) analysis based on the meta-analysis [13] and our previous experiment [20].

### 1.1. Predictions Based on the Adaptive Control Hypothesis and Dual Mechanisms Theory

As discussed previously, there are a few conditions in which the adaptive control hypothesis generates different predictions from those drawn based on the dual mechanisms theory of cognitive control. In the current experiment, we test these predictions using nine regions of interest identified by a meta-analysis on bilingual language control [13] and employed in our previous RITL investigation of bilingual language control mechanisms [20]. Below, we summarize where applicable, the distinct predictions each theory makes based on the current experimental design.

### 1.2. The Role of the Basal Ganglia Nuclei

One of the largest differences in predictions of the two frameworks tested herein stems from their descriptions of the role of the basal ganglia nuclei. Specifically, the adaptive control hypothesis proposes that the basal ganglia are involved in language switching or selection conditions; whereas the dual mechanisms framework proposes that the basal ganglia work in concert with the lateral prefrontal cortex to maintain the goal necessary to execute proactive control. Thus, the adaptive control hypothesis predicts that in the current experiment, greater basal ganglia activation should be observed during language switching trials as opposed to language repeat trials, presumably at the earliest point at which participants know in which language the trial will be executed (for cues during cued trials and during execution for non-cued trials). In contrast, the dual mechanisms theory predicts that the basal ganglia should be more active during cued trials, beginning at the moment of the cue and being maintained throughout execution, irrespective of whether language switches or repeats. This activation should be accompanied by increased activation in lateral prefrontal cortical areas, proposed to maintain the target language for proactive preparation.

### 1.3. The Role of Medial ACC/Pre-SMA ROIs

According to both frameworks, the ACC functions to detect, monitor and/or resolve conflict. The adaptive control hypothesis additionally discusses evidence that such mechanisms are particularly called upon in dual-language conditions, and that the Pre-SMA is likely involved in preparing and monitoring speech output specifically [11]. Thus, the adaptive control hypothesis predicts that the greatest Pre-SMA activation in the current experiment should be observed during sub-vocal task execution phases, and that this activation may be greater when languages switch from trial to trial than when they repeat. The dual mechanisms theory specifies the role of the ACC in triggering reactive control mechanisms. Thus, it also predicts that greater ACC activation should be observed during task execution, but in particular for trials in which no proactive language cue was given, and in individuals who do not engage language control mechanisms proactively. 

### 1.4. The Role of the Lateral Prefrontal ROIs

To the best of our knowledge, the dual mechanisms framework does not propose separate roles for the left or right lateral frontal cortices, nor for the different regions in the left prefrontal cortex in the current experiment in proactive control [26]. Thus, the dual mechanisms framework predicts that each of these regions will come online for cued trials in tandem with the basal ganglia, and will remain active throughout the task. The adaptive control hypothesis, however, suggests that the right frontal lobes form part of a circuit with the thalamus that functions to detect salient cues [11]; whereas the left inferior frontal cortex is proposed to be involved in response selection and inhibition. According to the adaptive control hypothesis, the right prefrontal ROI in the current experiment should be most active during cued trials over the language and rule cue phases, which provide salient information about the task at hand. The left inferior frontal ROI, on the other hand, should be most active during the execution phase of all trials, which forms the point at which the participant has all of the instructions for a trial and must select a response accordingly.

### 1.5. The Role of the Medial Temporal ROI 

The left temporal lobe is not part of the bilingual language control network outlined in the adaptive control hypothesis. In contrast, the dual mechanisms theory suggests that memory retrieval processes involving the temporal lobes may also reflect the triggering of reactive control mechanisms [26]. Thus, the dual mechanisms theory predicts that higher temporal lobe activation will be observed during the execution of non-cued tasks, or for tasks in which individuals did not choose to engage proactive control mechanisms. 

## 2. Materials and Methods

### 2.1. Participants

Seventeen right-handed Spanish–English bilinguals (8 female), aged 18–21 years, were paid for participation in the current study. Participants were required to be highly proficient in both languages, as assessed through grammatical proficiency tests, and to have learned both languages before the age of seven. All participants were healthy, with no history of developmental or neurological disorders. All participants provided informed consent, consistent with the protocols approved by the University of Washington’s Institutional Review Board. Participants’ language profile information is summarized in Table 1.

### 2.2. Materials

#### 2.2.1. Rapid Instructed Task Learning (RITL) Paradigm 

The RITL paradigm used in the current experiment consisted of 48 total trials, 24 English and 24 Spanish. Each trial contained one of two morpho-syntactic verb manipulations in equal numbers; third-person singular present conjugation or third-person plural present conjugation. The total of 48 trials were divided into six blocks of eight trials each, and each block contained equal proportions of English and Spanish trials and singular and plural verb conjugations. The trials were presented in pseudorandomized order, such that from trial to trial, languages switched 50 percent of the time (21 trials) and repeated 50 percent of the time (21 trials), with the remaining 6 trials being the first trial in each block. Across blocks, proactive and reactive control was manipulated. Participants were informed at the beginning of each block whether the trials within it would be cued (with target language presented in advance) or non-cued (with target language *not* presented in advance). The resulting three proactive, cued-language blocks (A) and three reactive, non-cued language blocks (B) were presented either in ABBAAB or in BAABBA order, counterbalanced across participants. 

Consistent with previous research on bilingual language control [20,25], each trial involved the presentation of information across four phases. The first “Prepare Target Language” phase consisted of two flags: the national flag of the Great Britain as a cue for English, and the national flag of Spain, as a cue for Spanish. During cued blocks, one of the two flags was presented in color to indicate which language would be used in the upcoming trial. In the non-cued blocks, both national flags were presented in grey, indicating that it was unknown which language would be in use in that trial. This condition was labeled as non-informative cues. The second “Select Rule” phase of the task involved the presentation of one of two morpho-syntactic verb conjugation rules: third-person *singular* present tense or third-person *plural* present tense. Singular present tense was indicated with a single stick figure, and plural present tense was indicated with two stick figures. The third “Execution” phase of the experiment involved the presentation of an infinitive verb in either English or Spanish. During the non-informative cued language blocks, this was the first indication participants had regarding in which language to execute the trial. During informative cued blocks, the language that the verb appeared in always matched the language indicated by the flag cue. Finally, a “Response Verification Probe” was presented to verify that participants had executed the correct rule subvocally. Half of the response probes were correct (matched the language and rule conjugation indicated by the trial) and the other half were false, consisting equally frequently of errors in target language, in conjugation rule, or in both target language and rule. A schematic of the paradigm is depicted in Figure 1.

All verbs selected were regular in conjugation in both Spanish and English. The average frequency in rank was 1125.25 (top 0.004%) with a standard deviation of 1130.74 (cf. median is 639 with minimum 73, and maximum 4265) [27]. Translational equivalents were presented across lists and across participants, not within lists. Sample stimuli for Spanish and English trials are listed in Table 2.

#### 2.2.2. Handedness Questionnaire 

Participants’ handedness was assessed using the Oldfield Handedness Inventory [28]. In the survey, participants were asked to rate whether they used left, right or both hands when they do 10 tasks. Handedness is then calculated using the ratio between right- greater than left-handed responses over right plus left-handed responses.

#### 2.2.3. English Proficiency Measure 

Participants’ English proficiency was measured using the English Grammatical Proficiency Test. The test is a subtest of the “Examination for the Certificate of Proficiency in English” developed at the University of Michigan [29]. Participants were asked to answer 20 multiple choice English grammatical questions; the assessment was untimed.

#### 2.2.4. Spanish Proficiency Measure 

Participants’ Spanish proficiency was assessed with the Spanish Grammatical Proficiency test. This test is a subtest of the standardized Spanish grammar proficiency test issued from the ministry of Spanish education for Diplomas in Spanish as a Foreign Language [30]. The assessment contains 20 multiple choice questions and 20 fill-in-the-blank questions, and was administered without a time limit. 

#### 2.2.5. Bilingual Language Experience Questionnaire 

Participants’ bilingual language experience and proficiency were assessed using a modified version of the Language Experience and Proficiency Questionnaire (LEAP-Q) [31]. The LEAP-Q asks participants to self-report their language comprehension, production, and reading proficiency, and also asks explicit questions about their backgrounds and experience with each language. This test has previously been used to investigate individual differences in bilingual language profile and experience (e.g., [20]).

## 3. Procedure

### 3.1. Behavioral Testing Session

The behavioral testing session was completed first, with the fMRI session following within two days. The behavioral session included completion of the Edinburgh Handedness Inventory [28] and LEAP-Q survey [31], plus the two proficiency tests [29,30]. Following completion of these tasks, participants completed an RITL practice run. Each participant completed 12 practice RITL trials composed of 2 blocks: informative cued language block (6 trials) and non-informative cued block (6 trials). In the practice trials, unlike the test trials in the scanner, participants were able to learn how they were performing through explicit feedback on response times and accuracy. Practice runs ensured that participants understood the task and could successfully perform it in the scanner. The total behavioral testing session took approximately one hour.

### 3.2. fMRI Data Acquisition 

Data were collected using a 3.0 T Philips Achieva scanner at the Integrative Brain Imaging Center operated by the University of Washington. The study was performed with a gradient echo planar pulse sequence with TR = 2000 milliseconds, TE = 25 milliseconds, a 79° flip angle and field of view = 240 mm × 240 mm. Thirty-eight oblique-axial slices were imaged in an ascending order, and each slice was 3-mm thick aligned to the anterior commissure-posterior commissure with no gap. The acquisition matrix was 80 × 80 with 3 × 3 × 3 mm voxels. This typically constitutes full coverage of the cerebrum. In the neuroimaging analysis procedures, any predefined region of interest that was not completely covered in all participants was excluded.

### 3.3. RITL Paradigm Presentation 

As is typical of RITL paradigms, the first three phases were self-paced and the Response Verification Probe phase was experimenter paced to ensure that participants had executed the rule during the execution phase. Participants were asked to press a button as soon as they encoded the instructions during the Prepare Target Language and Select Rule phases. During the Execute phase, participants pressed a button after they had conjugated the presented verb according to the instructions previously specified. If a button press was absent within 8 seconds during Prepare Target Language, Select Rule, or Execute phases, the trial “timed out” and automatically proceeded to the next phase. As is typical with RITL paradigms, the Response Verification Probe was only presented for 2000 milliseconds to encourage participants to generate the correct answer quickly. When presented with the Response Verification Probe, participants responded YES for correct or NO for incorrect answers. The button box with the hand corresponding to the position of the YES or NO labels on the screen was given. The position of the response labels on the screen was counterbalanced across participants. Accuracy to the Response Verification Probe was used to determine which trials were to be analyzed. Imaging acquired for incorrect trials was not analyzed.

To assess neural responses to the three critical task phases, each phase was separated from one another by delays with randomly varied durations between two and eight seconds, according to an exponential distribution [32]. The purpose of these delays is to reduce the collinearity between phases, and allowed for better estimation of the brain activity corresponding to each phase.

### 3.4. fMRI Data Processing

#### 3.4.1. fMRI Preprocessing

The data were preprocessed using SPM8 (Wellcome Trust Centre for Neuroimaging, Cambridge, UK). Functional volumes were corrected for slice timing acquisition, realigned to the first image within each run, normalized to the Montreal Neurological Institute (MNI) template, resampled to 2 mm^3^ voxels, and smoothed using an 8 mm Gaussian kernel.

#### 3.4.2. ROI Analyses

To best integrate our results with previous literature [20], which was based on a meta-analysis of bilingual language control [13], nine spherical regions of interest (ROIs) were used for ROI analyses. As in our previous study [20], all of the ROIs had 8 mm radii, with the exception of three: the Pre-SMA radius was adjusted to 10 mm to cover both hemispheres, and the left and right BG (caudate) ROI spheres were reduced to 6 mm to prevent them from extending into functionally distinct neighboring regions. The size, MNI coordinates of the centroids, and corresponding Brodmann’s areas (where applicable) of the nine ROIs used herein are listed in Table 3.

Summary statistics for the ROI analyses were generated by averaging across the parameter values (i.e., beta weights) of all voxels within the ROI. Summary statistics were calculated independently for each combination of ROI, subject, cue type (informative cued, and non-informative cued), switching condition (switched versus repeated language), and over each of the three critical task phases (Prepare Target Language, Select Rule, Execute). The data were then analyzed separately for each ROI using a 2 (cue type) × 2 (switch condition) × 3 (task phase) repeated measure analysis of variance (all effects were within-subject). All main effects and interactions were reported if they reached a significance level of *p* < 0.05, as these analyses were conducted based on hypotheses and ROIs defined a priori. False discovery rate (FDR) corrections for multiple comparisons are also reported for completeness. 

#### 3.4.3. Individual Differences Analyses

To explore the neurocognitive effects associated with proactive and reactive control, we conducted a series of correlational analyses with the following aims: (1) To identify the proactive control networks, the proactive cuing effects (activation to informative cues > activation to non-informative cues) were computed across the two caudate ROIs and correlated with the proactive cuing effects in each of the other four lateral prefrontal ROIs. The logic behind this analysis is that individuals that use proactive control strategies should, according to Braver [26], show increases in the lateral prefrontal cortex with accompanied increases in dopaminergic circuits. In contrast, individuals who do not recruit proactive control mechanisms should show little or no differences between informative and non-informative cues; (2) To identify the reactive control networks, controlled execution effects (activation during execution of non-informative cued trials > activation during execution of informative cued trials) were computed in the ACC and the temporal lobe, and correlated with each of the other seven ROIs. The logic behind this analysis resembles the former, namely that according to Braver [26], reactive control should result in a transient rise of activation during the execution phase, and this activation should be accompanied by either associative memory (temporal lobe) or conflict detection (anterior cingulate) mechanisms. Hence, reactive controllers should have smaller differences during execution of informative cued and non-informative cued trials in these areas than should proactive controllers. (3) To investigate the relation between proactive and reactive control, regions identified as part of the proactive control network through analysis one will be correlated with regions identified as part of the reactive control network in analysis two. Cuing effects in these regions should be anticorrelated. (4) To investigate the cognitive correlates of patterns of proactive and reactive control, cuing effects in the proactive and reactive control networks (identified through the first two analyses) will be correlated with cuing effects in response times. 

### 3.5. Behavioral Data Analysis

An angular transformation was performed in accuracy data since the distribution of accuracy was not normal. Incorrect responses in Probe phases were excluded from subsequent analyses. Out of three phases (i.e., Prepare Target Language, Select Rule, and Execute), response times of Execute phases following non-informative cues versus informative cues were compared to test the impact of cues. Response times that were three standard deviations above or below an individual participants’ mean were treated as outliers and excluded from response time analysis, resulting in the exclusion of 0.85% of the data. The time-out responses were replaced with the maximum time, 8000 ms for the analyses. This constituted 3.43% of the data. A 2 (switch and repeat) × 2 (cue and non-cued) × 3 (Prepare TL, Encode RL, and Execute) mixed effects models analysis was conducted on response times.

## 4. Results

### 4.1. Behavioral Results

Mean accuracy across participants was 97.06% with a standard deviation of 1.61%. Because participants were highly accurate, behavioral analyses were run only on response times (to correct trials), after outliers defined as three standard deviations above and below the means were removed. The mean response times for each condition are listed in Table 4. A 2 (cue present or absent) × 2 (switch versus repeat) × 3 (task phase) repeated measures analysis of variance (ANOVA) of mixed effects models revealed the main effects of cuing (*F*(1, 16) = 5.323, *p* = 0.023) and task phase (*F*(2, 32) = 384.516, *p* < 0.001). These results were modified by a significant cuing x task phase interaction (*F*(2, 32) = 9.210, *p* = 0.001). Follow-up analyses showed that, consistent with the broader proactive control literature and our predictions, informative cued trials resulted in marginally faster execution phase response times (*t*(16) = 1.948, *p* = 0.052), whereas response times during preparation were not significantly different for informative cued and non-informative cued blocks (Prepare Target Language: *t*(16) = 0.058, *p* = 0.954). No main effects or interactions with switching were found (*p* = 0.686). 

### 4.2. Region of Interest (ROI) Analyses

**Figure 2:**
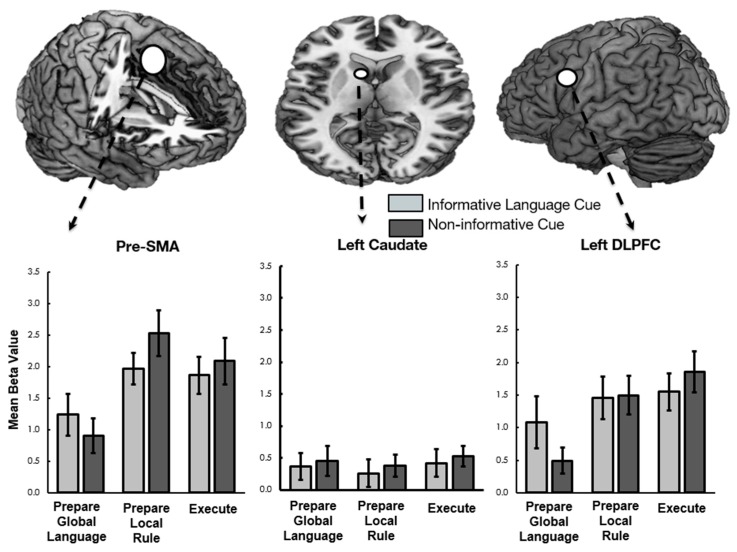
Mean beta weights extracted across the three task phases from three regions (pre-SMA, left caudate, left DLPFC).

Parallel 2 (cue present or absent) × 2 (switch versus repeat) × 3 (task phase) ANOVAs were also conducted on each bilingual language control ROI. Consistent with our previous research, a main effect of task phase was observed in left lateral frontal ROIs including DLPFC, lateral orbitofrontal cortex (BA 47), and IFG (BA 44), in the right precentral gyrus, and in the medial Pre-SMA (see Figure 2). Follow-up analyses revealed that each of these areas had greater activation during execution than encoding, consistent with the Execution Network described in our previous work [20].

Of interest to the current study, results revealed a significant interaction between cuing and task phase, in both in the Pre-SMA and the left DLPFC at the group level. Follow-up comparisons demonstrate that these regions were more active during cued than non-cued Prepare Target Language phases and were correspondingly less active during the rule (Pre-SMA only) and execution (both) phases (See Figure 2). The remaining four ROIs (ACC, bilateral caudate, and left middle temporal gyrus) were not modulated by our conditions of interest. *F*-statistics and follow-up paired *t*-statistics, including corrections for multiple comparisons, are reported in Table 5. 

### 4.3. Individual Differences Analyses

#### 4.3.1. Proactive Control Networks

**Figure 3:**
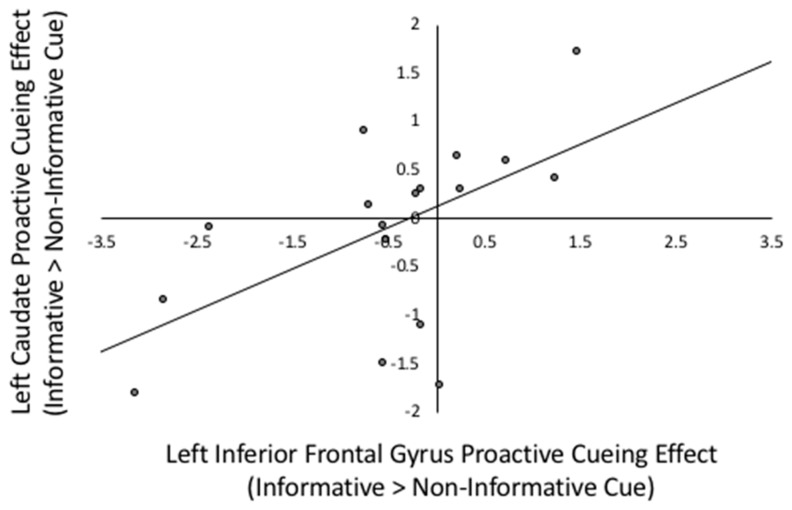
Scatterplot between left IFG proactive cueing effect and left caudate proactive cueing effect. Proactive cueing effect is the beta weight difference between informative cued > non-informative cued in Prepare Target Language phases, *r*(17) = 0.563, *p* = 0.019.

Consistent with the neural mechanisms described by Braver [26], the proactive cuing effect (informative > non-informative cues) in the left caudate nucleus was positively correlated with the proactive cuing effects in three out of four lateral prefrontal regions of interest (ROI) including the left DLPFC (*r*(17) = 0.561, *p* = 0.019), IFG(*r*(17) = 0.563, *p* = 0.019) and the right precentral gyrus (*r*(17) = 0.605, *p* = 0.010) ROIs, as well as with the right caudate nucleus (*r*(17) = 0.682, *p* = 0.003). The right caudate nucleus was only significantly correlated with cuing in the left DLPFC (*r*(17) = 0.559, *p* = 0.020). Each of these results was significant after the FDR multiple comparison correction was applied (uncorrected *p* < 0.023). To illustrate, the relations between cueing effects in the left IFG and left caudate nucleus are depicted in Figure 3. 

#### 4.3.2. Reactive Control Networks

**Figure 4:**
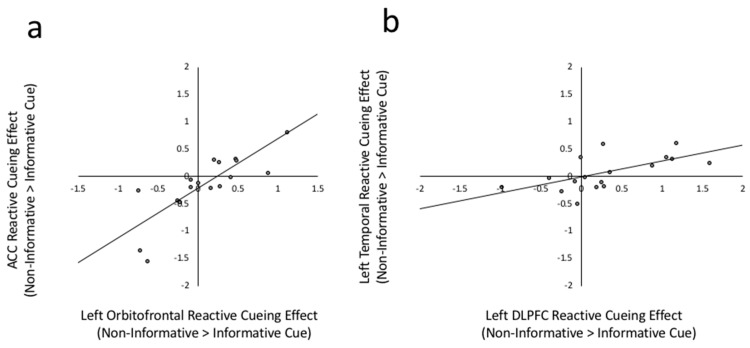
(**a**) Scatterplot between left Orbitofrontal reactive cueing effect and ACC reactive cueing effect. Reactive cueing effect is the beta weight difference between non-informative cued > informative cued in Execute phases, *r*(17) = 0.814, *p* < 0.001. (**b**) Scatter plot between left DLPFC reactive cuing effect and left temporal reactive cueing effect, *r*(17) = 0.614, *p* = 0.009.

The reactive cuing effect (execution of trials preceded by non-informative cues > execution of trials preceded by informative cues) in the ACC was strongly positively correlated with reactive cuing effects in the left orbitofrontal ROI (*r*(17) = 0.814, *p* < 0.001) as well as in the Pre-SMA (*r*(17) = 0.518, *p* = 0.033). Patterns of connectivity to the other candidate reactive control mechanism, the left temporal ROI, were quite different. Specifically, the reactive cuing effect in the left temporal ROI positively correlated with the reactive cuing effect in the lateral prefrontal regions including the left DLPFC (*r*(17) = 0.614, *p* = 0.009), the left IFG (*r*(17) = 0.523, *p* = 0.031) and the right precentral gyrus (*r*(17) = 0.588, *p* = 0.013) ROIs. The correlations between the ACC and Pre-SMA and between the left temporal and left IFG did not survive the FDR correction level of uncorrected *p* < 0.014. To illustrate, Figure 4 depicts the relation between reactive cueing effects in the ACC and left orbitofrontal ROI (Figure 4a) and between the left medial temporal and DLPFC regions (Figure 4b). 

### 4.4. Relating Proactive to Reactive Control Regions

The relation between proactive control (activation during informative > non-informative cues) and reactive activation patterns (activation during execution of non-informative > informative trials) was generally negative. Specifically, the cueing effects of three lateral prefrontal regions, DLPFC, IFG, and the right precentral gyrus correlated with the reactive execution effects in distributed ROIs. Left IFG cueing effect correlations were strongest and most distributed, reaching significance with the reactive execution effects in five out of the nine ROIs including the left IFG (*r*(17) = −0.697, *p* = 0.002), orbitofrontal (*r*(17) = −0.631, *p* = 0.007), and DLPFC (*r*(17) = −0.764, *p* < 0.001), the right precentral gyrus (*r*(17) = −0.507, *p* = 0.038), and the Pre-SMA (*r*(17) = −0.754, *p* < 0.001). Cuing effects in the left DLPFC negatively correlated with reaction execution effects in the left DLPFC (*r*(17) = −0.549, *p* = 0.023), the ACC (*r*(17) = −0.535, *p* = 0.027), and the right caudate (*r*(17) = −0.584, *p* = 0.014). The right precentral gyrus (*r*(17) = −0.507, *p* = 0.038), and cueing effects in the right precentral gyrus ROI were negatively correlated with reactive execution effects in the left DLPFC (*r*(17) = −0.628, *p* = 0.007) and the right precentral gyrus (*r*(17) = −0.528, *p* = 0.029) ROIs. All of these effects survived FDR correction which corresponded to the uncorrected *p* < 0.041.

### 4.5. Relating Proactive and Reactive Control Networks to Cognitive Demands

Neither proactive nor reactive control effects were reliably predictive of behavioral response times, although the proactive cuing effect in the left caudate approached significance with the cueing effect observed during the probe verification phase (*r*(17) = 0.43, *p* = 0.087). 

## 5. Discussion

The results of the current experiment, which adopted a dual mechanisms of cognitive control framework, extend our understanding of bilingual language control in several ways. First, consistent with the adaptive control hypothesis [11], we found that varying the conditions under which bilinguals control their language systems by manipulating the presence or absence of linguistic cues altered both the behavioral and neural responses. Consistent with previous research on proactive cuing in bilingual language use, [17,18,19], we found behavioral evidence that the presence of preparatory language cues facilitated subsequent execution of morphosyntactic rules. To the best of our knowledge, however, this is the first study to link this effect to patterns of neural activation. At the group level, a cue by task phase interaction was reflected by patterns of activation in the DLPFC and Pre-SMA ROIs (Figure 2), although only the former survived corrections for multiple comparisons. 

**Figure 5:**
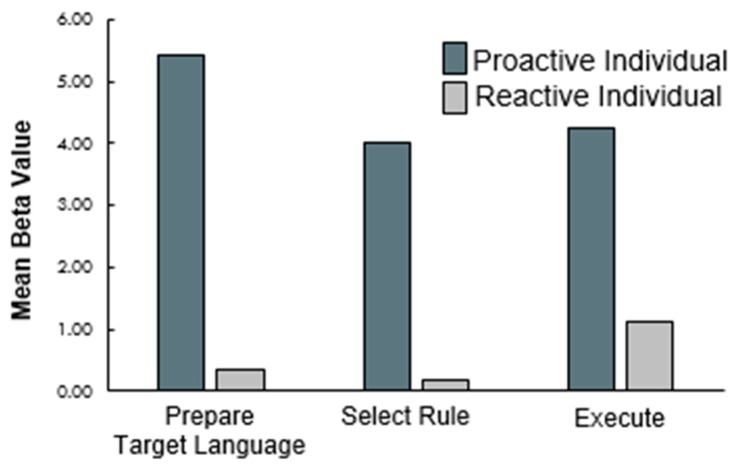
Two individuals’ beta weights extracted across the three task phases in cued trials.

Second, although the current analyses were conducted on a relatively small sample of bilinguals, the exploration of individual differences reported herein provides important modifications to our understanding of the neural basis of bilingual language control. Specifically, the Pre-SMA, DLPFC, IFG, and orbitofrontal areas all showed increasing patterns of activation across task phases at the group level in current and previous research [20]. Seo et al. [20] discussed this increase in activation as possibly arising from the accumulation of information in working memory. The addition of non-informative cued trials in the current experiment allowed us to explore individual sensitivity to the information presented in each of these phases. The results suggest that, consistent with the dual mechanisms framework, two distinct patterns of activation emerge across individuals. The first, proactive control pattern, shows early and sustained activation in the face of proactive global language cues, or the lack thereof in the case of IFG conflict monitoring. The second, reactive control pattern, shows late and transient activation during reactive task execution. When these patterns are summed across individuals, the averaged data reflect patterns that gradually increase across tasks. In fact, this pattern of activation is not commonly seen at the individual level. To illustrate, Figure 5 depicts patterns of DLPFC activation across informative cued task trials in two individuals. The first individual (in dark grey) shows a proactive pattern of activation and the second individual (in light grey) shows a reactive pattern of activation. Critically for future research, the adaptive control hypothesis also suggests that such individual differences in bilingual language control may arise because of stable differences in the contexts in which bilinguals speak. The relatively small and homogenous sample of bilinguals studied herein does not allow us to explore whether or not differences in bilingual language use correlate with differences in these patterns of activation. Similarly, the dual mechanisms framework suggests that both task variables and individual cognitive variables might determine whether a person deploys proactive or reactive control strategies. Future research measuring working memory capacity, for instance, in bilingual individuals could explore these claims more completely. Below, we provide an integrated summary of what these results contribute to our knowledge of the neural bases of bilingual language control, based on regions of interest.

### 5.1. Understanding the Role of the Lateral Prefrontal Cortical Regions in Bilingual Language Control 

The current study compared patterns of activation across four lateral prefrontal ROIs, the left IFG, DLPFC, orbital frontal, and the right precentral gyrus, which have been widely implicated in bilingual language control [12,20]. As discussed in the introduction, to the best of our knowledge, the dual mechanisms framework does not make specific predictions about how these regions might differ from one another; whereas the adaptive control hypothesis does. Three of these four regions, the IFG, DLPFC, and the right precentral gyrus, show patterns consistent with the dual mechanisms framework in that the extent to which they were more active to informative target language cues was correlated with activation in the dopaminergic basal ganglia nuclei. The orbitofrontal cortex, on the other hand, showed a pattern of activation more in line with reactive control or conflict monitoring, correlating strongly with reactive task execution in the ACC (Figure 4a).

The adaptive control hypothesis, however, suggests that the right precentral gyrus is involved in salient cue detection or inhibitory control processes whereas the DLPFC and IFG are involved in the control of both global and local interference, possibly through response selection [3]. Although the general patterns of these three regions with respect to individual differences in proactive and reactive control did not differ, the overall direction of their responses at the group level did differ in an interesting way. First, consistent with our previous work and with the adaptive control hypothesis, the two left prefrontal ROIs showed a general increase in activation from global to local task instruction phases through execution; whereas the right precentral gyrus did not ([21]: See Figure 2). In the current experiment, the right precentral gyrus was equally active for informative and non-informative cues at the group level, but was not engaged in task execution. In our previous research, the right precentral gyrus remained consistently activated across task phases and execution. Across experiments, the consistent engagement of the right precentral gyrus for global language selection and local rule selection phases suggests that the right precentral gyrus may be engaged either in the process of detecting salient cues, which are presented at each phase of the trial, or in some kind of general inhibitory process. It is unclear why, at the group level, the right precentral gyrus did not show differential responses to informative versus non-informative target language cues; however, individual differences analyses suggest that this was the case for some individuals and not for others. We see this as an interesting avenue for future research.

The current experiment replicates and extends our previous research on the role of the left lateral frontal regions in bilingual language control [20]. Both the current and previous research demonstrates that left lateral frontal regions show increased activation from global language preparation phases, to local rule selection phases, with the highest activation during task execution. The current experiment extends these results by showing that these regions are differentially sensitive to global language cues. Specifically, the DLPFC showed responses that are consistent with either a working memory or goal-maintenance view of bilingual language control. As evidenced by the significant cue by phase interaction (Figure 2), DLPFC activation was reliably greater to informative as opposed to non-informative cues and was subsequently less active in informative cued versus non-informative cued trial execution. In contrast, the IFG showed a pattern of responding that is more consistent with conflict management or response selection processes. Although the main effect of cueing was not significant (*p* = 0.087), IFG activation was generally greater for trials that began with non-informative cues, both during cuing and execution. As there was no specific instruction to hold in mind during non-informative cues, this pattern of responses suggests that the IFG responds in an anticipatory way to upcoming response conflict. The left orbitofrontal cortex was generally not sensitive to global language cueing at the group level.

**Figure 6:**
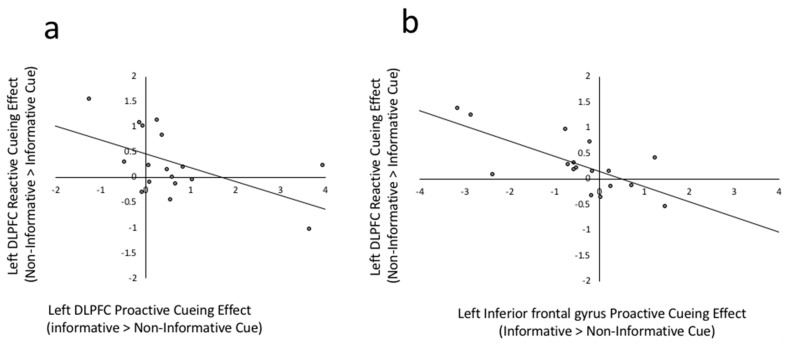
(**a**) Scatterplot between left DLPFC cueing effect and left DLPFC reactive cueing effect, *r*(17) = −0.549, *p* = 0.023. Proactive cueing effect is the beta weight difference between informative cued > non-informative cued in Prepare Target Language phases. Reactive cueing effect is the beta weight difference between non-informative cued > informative cued in Execute phases. (**b**) Scatter plot between left IFG proactive cuing effect and left IFG reactive cueing effect, *r*(17) = −0.697, *p* = 0.002.

Further evidence for a dissociation between the roles of the left DLPFC and IFG can be seen in the individual differences results. For example, Figure 6 depicts the relation between proactive and reactive cueing effects in the DLPFC (Figure 6a) and IFG (Figure 6b). Consistent with a dual mechanisms framework, the cueing effects in DLPFC were generally positive (Figure 6a, right of zero on X axis) and the extent to which an individual increased activation in DLPFC for informative cued trials carried over to execution, resulting in a negative reactive execution effect (non-informative cued > informative cued: below zero on y axis). In contrast, people who showed little difference in DLPFC activation to informative versus non-informative cues or showed greater activation to the latter (Figure 6a, left of zero of X axis) showed large reactive control effects during execution (above zero on the Y axis). Consistent with an interference management view, individuals who had a stronger IFG response to non-informative cues (Figure 6b, left of zero on X axis) also showed a stronger IFG response for non-informative trial execution (Figure 6b above zero on Y axis). Further evidence for dissociable roles of the DLPFC and IFG can be seen by the stronger and more widespread correlations between the proactive interference responses of IFG and the reactive execution responses of many of the regions in the bilingual language control network. This as an interesting avenue for future exploration. 

### 5.2. Understanding the Role of the Basal Ganglia Nuclei in Bilingual Language Control

A considerable amount of experimental [33], see review: [12], and theoretical [5,34,35,36] research has implicated the left caudate nucleus in bilingual language control. The current experiment extends this body of work in important ways. First, along with the meta-analysis of Luk et al., [13] and our previous research [20], we found that both left and right caudate nuclei are stably involved in bilingual language control. Specifically, across both experiments, we showed that the left and right caudate remained consistently active across global and local task preparation and execution phases. Although very little research to date has investigated laterality of language processes in the striatum, our results are inconsistent with the explanation of fronto-striatal laterality proposed by Crosson and colleagues [37], who suggested that right basal ganglia activation in the absence of right precentral gyrus activation reflected an inhibition of right frontal regions during a language production task. The correlation between cueing effects in the right caudate and the right precentral gyrus ROI did not reach significance, but it did approach significance and was in a *positive* direction [*r*(17) = 0.44, *p* = 0.077]. Also, the cuing effects in the left and right caudate were strongly positively correlated with one another [*r*(17) = 0.682, *p* = 0.003].

The results of the current research contribute two new pieces of evidence about the role of the bilateral caudate nuclei in bilingual language control: 1) that bilateral caudate nuclei are *not* differentially activated as a function of language switching versus repeating across trials, and 2) that individual differences in sensitivity to global language cues manifest via differences in activation between informative versus non-informative language cues, and correspond to individual differences in activation in prefrontal control regions and, to a marginal degree, differences in performance on informative cued versus non-informative cued trials (*p* < 0.10). These results can be viewed in light of the existing theories of the role of the basal ganglia in keeping track of the target language in use [20]. 

The results reported herein are consistent with the role of fronto-striatal signal biasing systems described in the conditional signal routing model of bilingual language control [5,23,24,38]. Specifically, the conditional signal routing model suggests that bilingual language control requires that the basal ganglia prioritize neural signals converging on the prefrontal cortex, according to the target language in use. The current study supports this explanation by showing that striatal responses to information about the target language are correlated with the responses of three target areas in the prefrontal cortex. It also extends this description by showing that the extent to which such signal prioritization is achieved proactively versus reactively varies across participants. 

### 5.3. Understanding the Role of the ACC/Pre-SMA Regions in Bilingual Language Control

The ACC and pre-SMA have been jointly associated with conflict monitoring in both the general cognitive control literature [39,40,41,42], and in the bilingual language control literature [43]. Both our previous and current research show differential patterns of responding in the ACC and pre-SMA. Specifically, Seo et al. [20] found that the ACC was most active during global language preparation, whereas the pre-SMA showed increased activation across the phases, with greatest activation during execution. Following that study, we proposed that ACC activation during global language preparation might have been particularly driven by trials in which the target language switched from one task to another. Results from the current experiment did not support this hypothesis. At the group level, ACC activation did not vary as a function of language switching, language cueing, or task phase. In contrast, the group level patterns of activation in the pre-SMA in both the previous and current research showed that activation increased across global language preparation, local rule preparation, and execution phases. A significant cue by phase interaction was observed in the current study, suggesting that proactive global language cues result in decreased pre-SMA activation during local rule selection and rule execution phases. Importantly, the presence of both informative and non-informative global language cues in the current study allowed us to investigate individual differences in the extent to which the ACC was sensitive to information about global language conflict either proactively or reactively. Here, the results showed that sensitivity to conflict during task execution was highly correlated between the ACC, Pre-SMA, and orbital frontal regions. 

Taken together, the pattern of results observed in the Pre-SMA more closely resembles that suggested by the adaptive control hypothesis (in conflict monitoring or controlling speech output) than does the pattern of results observed in the ACC. One review paper suggests a possible explanation for the dissociation of the ACC and Pre-SMA in our two experiments. Specifically, Hikosaka and Isoda (2010) propose that the ACC is sensitive to a negative feedback loop, whereas the Pre-SMA is more active in switching behavioral responses following a cue. Although no explicit feedback was given in either experiment, it is possible that the intersection between variability in task structure (e.g., when target language information was provided) and individual differences in deployment of proactive versus reactive control resulted in differences in the timecourse over which individuals experienced feedback about response conflict (indexed by ACC activation). We see this as an interesting avenue for future research. 

### 5.4. Understanding the Role of the Middle Temporal Lobe in Bilingual Language Control

Although the left middle temporal lobe has been implicated as a bilingual language control region in meta-analyses of bilingual language control [13], it is not included in the network of regions described in the Adaptive Control Hypothesis. In contrast, the dual mechanisms framework suggests that associative memory processes in medial temporal regions may trigger reactive retrieval of relevant task goals. The results of the current experiment as well as our previous research, suggest that the middle temporal ROI was consistently activated across task phases, and in conditions with or without informative global language cues. Importantly, the consistent activation of middle temporal lobes in the current experiment suggests that its involvement in our previous study was not tied to the memory demands associated with retrieving the mapping between abstract task rules and linguistic cues, as it did not change with the more naturalistic cues employed herein. Additionally, individual differences results suggested that reactive control in the left temporal lobe (increased activation during execution of trials preceded by non-informative versus informative cues) was positively correlated with similar reactive cueing effects in the three lateral prefrontal regions that were associated with proactive control. This is largely consistent with the reactive control mechanism proposed in the dual mechanisms framework [26], suggesting that when an individual fails to maintain a task instruction or goal prior to task execution, the left temporal lobe co-activates with frontal regions in a manner that resembles memory retrieval. Thus, it is plausible that the observed stability of temporal lobe activation across task phases at the group level does not reflect the patterns observed in individuals, but rather the result of averaging participants who encode and hold information in memory early, with those who reactively retrieve task-rules during execution.

## 6. Conclusions

With the inclusion of informative and non-informative cues, the current study extends previous research on bilingual language control by disentangling the neural mechanisms associated with proactive and reactive mechanisms. The results suggest that, consistent with the broader literature on bilingual language control and cognitive control more generally, the availability of target language cues facilitates bilingual morphosyntactic rule execution and reduces activation in the DLPFC and Pre-SMA during execution. However, consistent with the dual mechanisms prediction, we observed that not all individuals capitalize on such cues, and future research is needed to understand the extent to which these individual differences relate to bilingual language experience, cognitive capabilities, task demands, or (most likely) a convergence of all three factors. 

## Figures and Tables

**Table 1:** Profile of bilingual participants.

L2 Age of Acquisition		Spanish Grammatical Proficiency		English Grammatical Proficiency	
Mean Years	Range	Mean Percent	Range	Mean Percent	Range
4.3 (0.43)	0–5	76.94 (2.98)	50–94	92.94 (1.66)	80–100

**Table 2:** Sample stimuli for Spanish and English trials.

Grammatical Rule	English		Spanish	
	Stimulus	Response	Stimulus	Response
Singular Present	TO WALK	WALKS	CAMINAR	CAMINA
Plural Present		WALK		CAMINAN

**Table 3:** Description of interested regions.

Region	Centroid MNI Coordinates			Brodmann Area	Radius (mm)
Left Caudate	−14	5	16		6
Right Caudate	14	5	16		6
Anterior Cingulate Cortex	0	32	24	33	
Pre-SMA	1	1	57	6	10
Left DLPFC	−44	13	29	46	8
Right Precentral	40	−9	32	6	8
Left Inferior Frontal	−48	15	11	44	8
Left Lateral Orbitofrontal	−31	18	−2	47	8
Left Middle Temporal	−48	−42	−6	37	8

**Table 4:** Average reaction times (ms) in task phase by informative and non-informative trial.

	Informative	Non-Informative	Switch	Repeat
Prepare Target Language	1165.94 (102.40)	1204.69 (129.90)	1151.19 (123.13)	1113.21 (104.50)
Select Rule	1447.30 (160.24)	1459.78(153.06)	1466.30 (174.07)	1486.88 (150.27)
Execute	2470.37 (278.79)	91.68 (327.47)	2667.49 (298.94)	2690.03 (307.84)
Probe	724.73 (29.26)	716.87 (28.47)	712.50 (28.75)	729.45 (29.60)

Note. Standard errors of means are indicated in the parentheses.

**Table 5:** Statistics from 2 × 2 × 3 repeated measures ANOVA and follow-up tests for cueing effect and task phases on ROIs.

**(A) Significant Main Effect of Task Phase**	***df***	***F***	***MSE***	***p***
Pre supplementary motor area	2	14.007	25.757	<0.001 *
Left dorsolateral prefrontal cortex	2	16.112	15.446	<0.001 *
Left inferior frontal gyrus	2	7.526	4.915	0.002 *
Left lateral orbitofrontal cortex	2	5.890	2.870	0.007 *
Right precentral	2	5.696	3.991	0.008 *
**(B) Follow-up Effect of Phase**	***Phase***	***MD***	***SEM***	***p***
Preparation				
Right Precentral gyrus				
	Prepare TL > Execution	0.416	0.171	0.027
	Select RL > Execution	0.423	0.104	0.001
Execution				
Pre supplementary motor area				
	Select RL > Prepare TL	1.176	0.181	<0.001
	Execution > Prepare TL	0.903	0.269	0.004
Left dorsolateral prefrontal cortex				
	Select RL > Prepare TL	0.690	0.168	0.001
	Execution > Prepare TL	0.914	0.188	<0.001
Left Inferior frontal gyrus				
	Execution > Prepare TL	0.537	0.154	0.003
	Execution > Select RL	0.287	0.113	0.021
Left lateral orbitofrontal cortex				
	Execution > Prepare TL	0.389	0.127	0.008
	Execution > Select RL	0.309	0.101	0.008
**(C) Interaction**	***df***	***F***	***MSE***	***p***
Pre-SMA	2	3.903	3.476	0.030
DLPFC	2	8.471	3.612	0.001 *

Note. The levels of each factor are as follows: Cueing (2): Cued, Non-cued; Switching (2): Switching, Repeating; Phase (3): Prepare Target Language (TL), Select Rule (RL), Execution. *, Asterisks in main effects and interactions represent significance according to false a discovery rate correction of *p* > 0.0081.

## References

[1] Bialystok E., Craik F.I., Luk G. (2012). Bilingualism: Consequences for mind and brain. Trends Cogn. Sci..

[2] Shin H.B., Kominski R. (2010). Language Use in the United States, 2007.

[3] Abutalebi J., Green D.W. (2016). Neuroimaging of language control in bilinguals: Neural adaptation and reserve. Biling. Lang. Cogn..

[4] Blanco-Elorrieta E., Pylkkänen L. (2018). Ecological validity in bilingualism research and the bilingual advantage. Trends Cogn. Sci..

[5] Buchweitz A., Prat C. (2013). The bilingual brain: Flexibility and control in the human cortex. Phys. Life Rev..

[6] Bialystok E. (2001). Bilingualism in Development: Language, Literacy, and Cognition.

[7] Costa A., Miozzo M., Caramazza A. (1993). Lexical selection in bilinguals: Do words in the bilingual’s two lexicons compete for selection?. J. Mem. Lang..

[8] Kroll J.F., Bobb S.C., Wodniecka Z. (2006). Language selectivity is the exception, not the rule: Arguments against a fixed locus of language selection in bilingual speech. Biling. Lang. Cogn..

[9] Kroll J.F., Stewart E. (1994). Category interference in translation and picture naming: Evidence for asymmetric connections between bilingual memory representations. J. Mem. Lang..

[10] Van Heuven W.J., Dijkstra T., Grainger J. (1998). Orthographic neighborhood effects in bilingual word recognition. J. Mem. Lang..

[11] Green D.W., Abutalebi J. (2013). Language control in bilinguals: The adaptive control hypothesis. J. Cogn. Psychol..

[12] Braver T.S., Gray J.R., Burgess G.C. (2007). Variation in Working Memory.

[13] Luk G., Green D.W., Abutalebi J., Grady C. (2012). Cognitive control for language switching in bilinguals: A quantitative meta-analysis of functional neuroimaging studies. Lang. Cogn. Proc..

[14] Kane M.J., Engle R.W. (2002). The role of prefrontal cortex in working-memory capacity, executive attention, and general fluid intelligence: An individual-differences perspective. Psychon. Bull. Rev..

[15] Locke H.S., Braver T.S. (2008). Motivational influences on cognitive control: Behavior, brain activation, and individual differences. Cogn. Affect. Behav. Neurosci..

[16] Bonfieni M., Branigan H.P., Pickering M.J., Sorace A. (2019). Language experience modulates bilingual language control: The effect of proficiency, age of acquisition, and exposure on language switching. Acta Psychol..

[17] Grainger J., Declerck M., Marzouki Y. (2017). On national flags and language tags: Effects of flag-language congruency in bilingual word recognition. Acta Psychol..

[18] Martin C.D., Molnar M., Carreiras M. (2016). The proactive bilingual brain: Using interlocutor identity to generate predictions for language processing. Sci. Rep..

[19] Woumans E., Martin C.D., Vanden Bulcke C., Van Assche E., Costa A., Hartsuiker R.J., Duyck W. (2015). Can faces prime a language?. Psychol. Sci..

[20] Seo R., Stocco A., Prat C.S. (2018). The bilingual language network: Differential involvement of anterior cingulate, basal ganglia and prefrontal cortex in preparation, monitoring, and execution. NeuroImage.

[21] Cole M.W., Laurent P., Stocco A. (2013). Rapid instructed task learning: A new window into the human brain’s unique capacity for flexible cognitive control. Cogn. Affect. Behav. Neurosci..

[22] Stocco A., Lebiere C., O’Reilly R.C., Anderson J.R. (2012). Distinct contributions of the caudate nucleus, rostral prefrontal cortex, and parietal cortex to the execution of instructed tasks. Cogn. Affect. Behav. Neurosci..

[23] Becker T.M., Prat C.S., Stocco A. (2016). A network-level analysis of cognitive flexibility reveals a differential influence of the anterior cingulate cortex in bilinguals versus monolinguals. Neuropsychologia.

[24] Stocco A., Prat C.S. (2014). Bilingualism trains specific brain circuits involved in flexible rule selection and application. Brain Lang..

[25] Seo R., Prat C.S. Investigating Local and Global Control Mechanisms in Bilingual Grammatical Processing. J. Exp. Psychol. Learn. Mem. Cogn..

[26] Braver T.S. (2012). The variable nature of cognitive control: A dual mechanisms framework. Trends Cogn. Sci..

[27] English Frequency List. https://en.wiktionary.org/wiki/Wiktionary:Frequency_lists.

[28] Oldfield R.C. (1971). The assessment and analysis of handedness: The Edinburgh inventory. Neuropsychologia.

[29] English Language Institute (2006). Examination for the Certificate of Proficiency in English: Information Bulletin.

[30] (1998). el Ministerio de Educacion, Diplomas de espa nol (DELE).

[31] Marian V., Blumenfeld H.K., Kaushanskaya M. (2007). The Language Experience and Proficiency Questionnaire (LEAP-Q): Assessing language profiles in bilinguals and multilinguals. J. Speech Lang. Hear. Res..

[32] Dale A.M. (1999). Optimal experimental design for event-related fMRI. Hum. Brain Mapp..

[33] Crinion J., Turner R., Grogan A., Hanakawa T., Noppeney U., Devlin J.T., Aso T., Urayama S., Fukuyama H., Stockton K. (2006). Language control in the bilingual brain. Science.

[34] Abutalebi J. (2008). Neural aspects of second language representation and language control. Acta Psychol..

[35] Friederici A.D. (2006). What’s in control of language?. Nat. Neurosci..

[36] Stocco A., Yamasaki B., Natalenko R., Prat C.S. (2014). Bilingual brain training: A neurobiological framework of how bilingual experience improves executive function. Int. J. Biling..

[37] Crosson B., Benefield H., Cato M.A., Sadek J.R., Moore A.B., Wierenga C.E., Gökçay D. (2003). Left and right basal ganglia and frontal activity during language generation: Contributions to lexical, semantic, and phonological processes. J. Int. Neuropsychol. Soc..

[38] Yamasaki B.L., Stocco A., Liu A.S., Prat C.S. Effects of Bilingual Language Experience on Basal Ganglia Computations: A Dynamic Causal Modeling Test of the Conditional Routing Model. Brain Lang..

[39] Duncan J. (2010). The multiple-demand (MD) system of the primate brain: Mental programs for intelligent behaviour. Trends Cogn. Sci..

[40] Garavan H., Ross T.J., Murphy K., Roche R.A.P., Stein E.A. (2002). Dissociable executive functions in the dynamic control of behavior: Inhibition, error detection, and correction. NeuroImage.

[41] Milham M.P., Banich M.T., Webb A., Barad V., Cohen N.J., Wszalek T., Kramer A.F. (2001). The relative involvement of anterior cingulate and prefrontal cortex in attentional control depends on nature of conflict. Cogn Brain Res..

[42] Hester R.L., Murphy K., Foxe J.J., Foxe D.M., Javitt D.C., Garavan H. (2004). Predicting success: Patterns of cortical activation and deactivation prior to response inhibition. J. Cogn. Neurosci..

[43] Abutalebi J., Della Rosa P.A., Ding G., Weekes B., Costa A., Green D.W. (2013). Language proficiency modulates the engagement of cognitive control areas in multilinguals. Cortex.

